# Smoking, Nutritional Status, and Their Associations with Hypertension and Hematological Disorders Among Hotel Workers: Implications for Indonesian Occupational Health Nursing

**DOI:** 10.3390/ijerph23040505

**Published:** 2026-04-15

**Authors:** Juli Dwi Prasetyono, Henny Permatasari, Agus Setiawan, Sigit Mulyono, Tantut Susanto, Muchtaruddin Mansyur

**Affiliations:** 1 Department of Nursing Science, Faculty of Nursing, Universitas Indonesia, Depok 16424, Indonesia; juli.dwi21@ui.ac.id (J.D.P.); hennyp232@gmail.com (H.P.); a-setiawan@ui.ac.id (A.S.); sigit@ui.ac.id (S.M.); 2 Department of Community, Family & Geriatric Nursing, Faculty of Nursing, Universitas Jember, Jember 68121, Indonesia; tantut_s.psik@unej.ac.id; 3 Department of Community Medicine, Faculty of Medicine, Universitas Indonesia, Central Jakarta 10430, Indonesia

**Keywords:** smoking, nutritional status, hypertension, leukocytosis, hematological disorders, occupational health, hotel workers, Indonesia

## Abstract

**Highlights:**

**Public health relevance—How does this work relate to public health issues?**

- Hotel workers who are older, overweight, and smoke have higher risks of hypertension and blood abnormalities in comparison with their peers; this represents a workplace based public health intervention need.
- The findings reinforce the role of occupational health nurses in smoking cessation, weight control, and routine monitoring programs to reduce disease burden among workers.

**Public health significance—Why is this work important to public health?**

- The findings can help in the prevention of chronic disease in workers by identifying smoking and overweight status as key risk factors for hypertension and blood disorders.
- The findings can be used to support occupational health nurses in delivering targeted workplace health programs to reduce modifiable risk factors and improve worker health.

**Public health implications—What are the key implications or messages for public health practitioners, policy makers, and researchers?**

- We recommend that high-risk workers are supported with targeted screening and lifestyle interventions to reduce the risk of preventable chronic diseases.
- Occupational health nurses should be empowered to lead health-promoting and risk-reduction programs in workplaces.

**Abstract:**

Smoking remains one of the leading preventable causes of chronic disease and premature workplace mortality worldwide. This study examined the association between smoking and nutritional status and hypertension and hematological disorders among hotel workers and occupational health nurses’ role in Indonesia. This cross-sectional study examined associations between smoking, nutritional status, and selected health outcomes among 366 hotel workers in Indonesia using routine medical check-up data. Logistic regression analyses were performed to assess the associations of smoking status and body mass index (BMI) categories with hypertension and hematological abnormalities (leukocytosis and anemia), after adjusting for age, gender, and job level. Older workers (40–69 years) and those categorized as overweight or obese had higher odds of hypertension than younger workers and those with normal BMI (ORs 2.63 and 1.37, respectively). Smoking was associated with a higher risk of leukocytosis (OR 0.395), reflecting increased risk among smokers due to variable coding. Older age and overweight status were strong predictors of hypertension, whereas smoking was associated with increased leukocytosis among hotel workers. These findings highlight the need for targeted OH interventions. Occupational health nurses should collaborate with management to strengthen WHP programs that encourage healthier lifestyles among employees.

## 1. Introduction

The intricate relationship between health and place among hotel workers, particularly those with hypertension and hematological disorders, is significantly influenced by occupational health factors, environmental stressors, and specific workplace conditions prevalent in the hospitality industry. These factors collectively contribute to the prevalence and severity of these health conditions [1,2,3,4]. Hotel workers, especially housekeepers, frequently face challenging working conditions that increase their risk for various health issues, including hypertension [5,6,7]. For instance, a study on hotel staff in Bali investigated the comparison of physical worker status, anthropometric status, and dyslipidemia toward the prevalence of hypertension [8]. This suggests that the demanding nature of their work, which often involves 8–9 h of physical activity per shift, could be a contributing factor. Such physical exertion, combined with other workplace stressors, can increase cardiovascular load. Research assessing cardiovascular load among hotel room cleaners found significantly higher mean average and peak heart rate, percentage heart rate reserve, metabolic equivalent, and energy expenditure during workdays compared to off-work days [9].

Hypertension and hematologic disorders represent critical health problems in hotel workers, with substantial evidence linking their prevalence and severity to modifiable risk factors such as smoking and nutritional status [10,11,12,13,14,15]. Understanding these complex relationships, particularly within specific working populations such as hotel employees, is crucial for developing targeted health interventions and improving occupational well-being [5,16,17]. Hypertension, defined as a persistent elevation in blood pressure above 140/90 mmHg [18,19,20], is a major global health challenge and a primary risk factor for cardiovascular diseases, including stroke and heart failure [18,19,20,21,22,23]. Its increasing incidence is often attributed to unhealthy eating patterns, sedentary lifestyles, and chronic stressors [10,24,25,26,27]. Smoking and nutritional status are consistently significant contributors to hypertension development and progression [10,11,15,26,28]. Smoking, for instance, has been directly associated with an increased risk of hypertension [13,22,29,30], primarily due to the harmful chemicals in tobacco smoke that induce chronic inflammation and endothelial dysfunction, impacting drug metabolism and exacerbating cardiovascular risks [14,18,31]. Nicotine acts as a vasoconstrictor, significantly increasing blood pressure [32]. Physiological mechanisms involve not only direct vascular damage but also the systemic impact of chronic inflammation and oxidative stress that propagates throughout the body [14,18,31,33,34,35,36].

Smoking is associated with increased hematocrit and may influence erythropoiesis and red cell parameters, including potential hyporesponsiveness to erythropoiesis-stimulating agents in certain workers [37,38,39]. Smoking also has a notable prevalence of 18.3% in this population, indicating its widespread contribution to health risks [40]. Nutritional status, which encompasses diet quality and body composition, also plays a pivotal role in BP regulation [10,20,26,41,42]. Diets high in energy, carbohydrates, fat, cholesterol, sugar, and salt, particularly instant foods, are strongly linked to hypertension [43,44]. Obesity, which is often a consequence of poor nutritional status, is consistently identified as a risk factor for hypertension [11,14,15,23,27,40,44]. The relationship between various dietary components and blood parameters is complex, with high sodium intake, certain fats, and high sugar consumption correlating with increased cardiovascular risks [41,43].

Hotel workers in mountainous areas represent a unique cohort of workers exposed to specific occupational hazards and psychosocial stressors that can further intensify these health risks [5,16,17,25]. For example, hotel room cleaners experience significantly higher mean and peak heart rates, heart rate reserve percentages, metabolic equivalents (METs), and energy expenditure during workdays compared to off-work days, indicating considerable cardiovascular load [9]. Psychosocial work stress, which is often prevalent in the hotel and catering industry due to factors such as shift work, is recognized as a risk indicator for impaired cardiovascular and psychological health [25]. Job stress has also been shown to influence the eating behavior of hotel employees, potentially contributing to poor nutritional status [45]. Furthermore, these workers may be exposed to ETS in hospitality venues, particularly in regions without comprehensive smoking bans, adding another layer of risk [14,46,47]. The role of occupational health nurses (OHNs) can be understood through their broader functions in workplace health and safety based on the occupational hazards among hotel workers. OHN could contribute to managing such psychosocial factors by promoting positive workplace environments and addressing emotional health [48] by integrating across individual, interpersonal, organizational, and policy levels to optimize health and well-being of hotel workers [49].

Smoking, suboptimal nutritional status, and occupational stressors can significantly heighten the risk of hypertension and potentially contribute to other systemic health issues, including hematologic disorders. In Indonesia, hypertension affected approximately 34% of adults (approximately 63.3 million people) and led to approximately 427,000 deaths in 2018, and the prevalence remains high at 29.2% in 2023 [50]. While specific research directly linking smoking and nutritional status to hematologic disorders among hotel workers is less abundant in the provided literature, smoking is known to affect hematologic parameters. Smoking, particularly hookah and tobacco, has been associated with polycythemia (an increase in red blood cell count) and hypoxia, which can contribute to hypertension and affect overall blood parameters [32]. Nutritional status also critically influences hematologic health, as evidenced by studies on patients with hematological malignancies, where nutritional alterations can occur after treatments such as chimeric antigen receptor T-cell therapy [51]. Therefore, chronic exposure to smoking and inadequate nutrition could compromise hematologic health in this vulnerable population. The broader understanding of cardiovascular load among hotel workers [9] and the impact of stressors on allostatic load [17] further support the need for comprehensive health assessments. The study was conducted in a mountain-area hotel complex, where colder temperatures, uneven terrain, and higher physical demands may increase physiological strain during daily tasks. These environmental conditions may interact with smoking and nutritional factors, making the workplace context relevant for interpreting workers’ cardiovascular and hematological outcomes. The high risk of occupational hazards and illnesses among hotel workers should be a serious concern for occupational health nursing practice. However, in Indonesia, OHN has not played a significant role in providing health services for workers. The studies’ findings indicate that OHNs, as paramedics, primarily serve as first responders during emergencies affecting hotel guests or visitors. In fact, there is a potential strategic role for OHNs to be involved in health promotion activities within the hotel environment, and disease prevention, including Hypertension control.

The studies concluded that the hotel workers’ health, particularly those with hypertension, is demonstrated to be affected by smoking habits and nutritional status, compounded by occupational factors inherent to their industry. While detailed direct links to hematologic disorders among this specific group require further dedicated investigation, the existing literature broadly supports the harmful effects of smoking and poor nutrition on both cardiovascular and hematological systems. Addressing these modifiable risk factors through targeted interventions and health promotion efforts is essential for mitigating adverse health outcomes in this important workforce [5]. Although previous studies have examined ergonomic and psychosocial risks among hotel workers, limited evidence exists on how modifiable lifestyle factors, such as smoking and nutritional status, are related to hypertension and hematological abnormalities in this occupational group. This gap is particularly notable in Indonesia, where routine biological health monitoring is uncommon. Addressing this gap is essential to inform occupational health nursing roles and develop targeted workplace health promotion strategies. Therefore, this study was conducted to investigate the relationship between age, gender, job level, smoking status, nutritional status, hypertension, and hematological disorders among hotel workers in Indonesia.

## 2. Materials and Methods

### 2.1. Study Population

This is an observational analytic study using a cross-sectional design. Data collection was conducted in April 2025. This research was conducted after obtaining approval for the implementation of research from the Ethics Commission of the Faculty of Nursing, University of Indonesia, with Number: KET-186/UN2. F12. D1.2.1/PPM.00.02/2025 on 6 February 2025.

This study used a census or total population sampling approach, in which all hotel workers were invited to participate. Of the 370 eligible employees, 366 agreed to participate and provided complete data. Therefore, the findings are representative of the hotel’s worker population. All the respondents in this study were from one hotel worker at SA Company, Ciater Subang, West Java, Indonesia. A census approach was used because the total number of hotel workers was relatively small (*N* = 370) and all employees were accessible to the research team. A census is preferable in settings where the target population is limited and fully reachable because it eliminates sampling error and ensures complete representation of the population. The independent variables in this study were personal factors (age, gender, and smoking habits) and job level (division). Meanwhile, the dependent variables were hypertension status and hematological disorders.

Respondents were eligible to participate if they met the following inclusion criteria: active employees at SA Company during the data collection period, aged > 18 years, provided complete information on key study variables, and available and present on-site during data collection. Exclusion criteria: Those who were absent, on leave, or no longer employed during the data collection period, had a known medical condition that could influence hematologic values (e.g., chronic blood disorders, acute infections), were pregnant due to physiological changes that affect hemoglobin and leukocyte levels, or did not provide informed consent were excluded from the study.

Data on personal factors (age, gender, job level, and smoking status) were obtained through a questionnaire. In this case, a questionnaire was also employed by instructing the respondent to answer the questions according to the circumstances experienced by the respondent. The research team collected personal variables (age, gender, job level, and smoking status) using a structured questionnaire. The questionnaire consisted of closed-ended items and was pilot-tested among 20 hotel workers to ensure clarity and content validity. Smoking status was assessed using a single binary question (‘Do you currently smoke cigarettes?” Yes/No’), while age, gender, and job level were self-reported. Trained data collectors administered the questionnaire during the medical check-up session.

Nutritional status can be calculated using the body mass index (BMI) with the formula BMI = weight (kg) ÷ (height (m)^2^), then compared to the Indonesian Ministry of Health Standard, categories: Underweight (<18.5); Normal (18.525.0); Overweight (25–27.0) and Obesity (≥27.0). Camry brand scales (Zhongshan City, China) were calibrated with an accuracy of 0.01 kg to determine body weight, and GEA brand microtoise (East Jakarta, Indonesia) with an accuracy of 0.1 cm were calibrated to determine height.

The incidence of hypertension was measured using a GEA brand sphygmomanometer to obtain blood pressure data, with assistance from nurses, and to diagnose hypertension in accordance with the American Heart Association (AHA) guidelines. According to the 2025 AHA/ACC guidelines, BP categories are defined as follows: Normal: <120/<80 mmHg; Elevated: 120–129/<80 mmHg; Hypertension: (Stage 1: 130–139/80–89 mmHg; and Stage 2: ≥140/≥90 mmHg). BP was measured once for each participant during the medical checkup using a calibrated GEA sphygmomanometer. Measurements were taken with the participant seated after at least 5 min of rest. This single reading was used to classify hypertension status following the 2025 AHA/ACC guidelines.

In this study, the term “hematological disorder” was used as an operational definition to refer to abnormalities in hematologic parameters identified through laboratory examination, including anemia and white blood cell count variations. This term does not imply that all included conditions represent primary hematologic diseases; specifically, leukocytosis is considered a physiological inflammatory response but is included in this category for analytical purposes. Leukocytosis was defined as a total leukocyte count greater than 11,000/µL based on the standard adult clinical reference ranges. Anemia was defined using sex-specific hemoglobin cutoffs (<13.0 g/dL for men and <12.0 g/dL for nonpregnant women). For analysis, each outcome was coded as 0 = normal and 1 = abnormal.

### 2.2. Statistical Analysis

Data were analyzed using univariate, bivariate, and multivariate methods. Bivariate analysis was performed using Spearman’s *t*-test with a significance level of 0.05. Univariate analysis was performed to present data on personal factors, and bivariate analysis was performed to analyze the relationship between personal factors, job level, smoking, and nutritional status using the Spearman correlation test. The Spearman correlation test was used to analyze the relationship between two variables, such as smoking habits and nutritional status, with a minimum ordinal data scale. Spearman’s rank correlation was used in the bivariate analysis because several independent variables, such as smoking status, nutritional status, and job level, were ordinal or non-normally distributed categorical variables. The Spearman correlation allowed us to explore the monotonic associations between these predictors and the binary health outcomes before conducting the multivariate logistic regression. This approach served as a non-parametric screening step to identify variables potentially associated with HTN and hematological disorders. Each variable with an ordinal data scale was tested for its relationship with the hypertensive status and hematological disorders (leukocytosis or anemia). Logistic regression analysis was conducted with smoking and nutritional status as the independent variables and hypertension status and hematological disorders as the dependent variables. Variables with a bivariate association with *p* ≤ 0.25 were initially considered for inclusion in the multivariate logistic regression models, following established confounder-screening recommendations. All eligible variables were entered into the preliminary models. Variables that showed multicollinearity (variance inflation factor > 10), produced unstable coefficients, or lacked theoretical relevance were removed in a stepwise refinement process. The final adjusted models in Tables 3 and 4 include only variables that met the statistical, conceptual, and collinearity criteria. This updated approach is now fully reflected in the tables reported. All analyses were performed using the Statistical Package for the Social Sciences version 26.

## 3. Results

### 3.1. Characteristics of Hotel Workers

Table 1 shows the personal worker factors, including age, gender, job level, nutritional status, smoking status, hypertension, and hematological disorders (leukocytosis and anemia). Most workers were middle-aged and predominantly male. Most were employed in operational divisions such as recreation/pool, food and beverage, and security. Overweight and obesity were common, and more than half of the workers were smokers. Hypertension and hematological abnormalities (leukocytosis or anemia) were also observed in a notable proportion of the workforce.

### 3.2. Relationship Between Age and Nutritional Status and Hypertension Among Hotel Workers

Associations between age and nutritional status and hypertension are shown in Table 2. In the unadjusted analysis, hypertension was significantly related with age from 40–69 years and overweight nutritional status (*p* < 0.05).

Table 3 presents the adjusted logistic regression results for the association between nutritional status and age. After controlling for gender, job level, and smoking habit, multiple logistic regression showed that overweight or obesity and age were significantly positively associated with hypertension among hotel workers. Workers aged 40–69 years had higher odds of hypertension (odds ratio [OR] = 2.63, 95% confidence interval [CI]: 1.61–4.33) than younger workers. Similarly, those classified as overweight had increased odds of hypertension (odds ratio [OR] = 1.36, 95% confidence interval [CI]: 1.13–1.66).

Table 4 presents the smoking status affecting leukocytosis after controlling for age, gender, job level, and nutritional status. Multiple logistic regression showed that smoking status (0 = smokers; 1 = non-smokers) was significantly positively associated with leukocytosis among hotel workers (B = −0.928; β = 0.395, 95% CI 0.188–0.834).

## 4. Discussions

This study found that older age and overweight status were associated with higher odds of hypertension, whereas smoking was associated with increased odds of leukocytosis among hotel workers. The logistic regression results indicate that older workers and those with overweight status were more likely to have hypertension, consistent with established evidence linking age and body weight to increased risk of hypertension. The associations observed in this study suggest that older age, overweight status, and smoking may contribute to a higher likelihood of hypertension and hematological abnormalities among hotel workers, without implying direct causation.

### 4.1. Relationship Between Age and Nutritional Status and Hypertension

Older workers and those with overweight status demonstrated higher odds of hypertension, highlighting the role of age-related physiological changes and excess body weight in elevating cardiovascular risk. The findings from the multivariate logistic regression analysis in the study indicated that individuals aged 40–69 years showed a higher propensity for hypertension (β = 2.63) compared to workers aged 40 years [52]. This observation aligns with broader epidemiological data indicating that age is a significant risk factor for hypertension [53]. Furthermore, overweight status was significantly associated with elevated hypertension levels (β = 1.36), consistent with the established links between nutritional status, obesity, and hypertension [26,52,54,55,56]. Obesity, in particular, is recognized to increase the risk of hypertension, often contributing to metabolic syndrome, which increases the risk of cardiovascular diseases [53,56,57].

### 4.2. Smoking and Hematological Abnormalities

Smoking status was associated with increased leukocytosis risk, reflecting well-established inflammatory responses to tobacco exposure. Smoking, nutritional status, and their implications for hypertension and hematological disorders, particularly leukocytosis, represent significant public health concerns, particularly within specific occupational groups such as hotel workers in Indonesia [23,26,52]. Hypertension is a prevalent non-communicable disease in Indonesia, with a prevalence of 34.1% across the population [58,59]. The incidence of hypertension is known to increase with age, affecting 31.6% of the 31–44 age group, 45.3% of the 45–54 age group, and 55.2% of the 55–64 age group. Lifestyle factors, including unhealthy dietary patterns, lack of physical activity, and smoking habits, are significant contributors to hypertension [10,26,60,61].

A critical finding of the study was the association between smoking and hematological parameters, specifically leukocytosis [52]. Smokers exhibited higher levels of leukocytosis (β = 0.39) than non-smokers. This is supported by other research, which indicates that smoking causes chronic, body-wide inflammation and significantly impacts hematological indices, including increased white blood cell counts [62,63,64]. The chemicals in tobacco smoke, which are distinct from nicotine, contribute to this inflammatory state and can lead to increased blood viscosity, primarily due to a raised hematocrit in smokers. Such hematological disturbances are also observed in conditions such as diabetes and hypertension, further compounding cardiovascular risks [64].

### 4.3. Role of Occupational Health Nurses and Implications for Worker Health

The demanding physical tasks and environmental conditions in the hotel setting may interact with individual lifestyle factors, underscoring the need for workplace-based screening and health promotion strategies. The hotel workers’ occupational context introduces unique considerations. Hotel employees often face challenging working conditions, including physically demanding tasks, long hours, and psychosocial hazards, which can predispose them to various health issues, including musculoskeletal disorders and increased stress [1,64,65]. These factors can interact with lifestyle choices, such as smoking and nutritional habits, to intensify health risks [5,17]. For instance, the “Healthy Worker Effect” suggests that employed populations may appear healthier than the general population due to selection bias, where healthier individuals are more likely to be hired and remain in demanding jobs. However, specific occupational exposures and lifestyle factors can still lead to significant health disparities even within this selected group.

These findings have substantial implications for occupational health. The high prevalence of smoking in Indonesia underscores the need for targeted interventions, where rates have remained largely unchanged despite various policies [66]. The combined effect of smoking and obesity on the onset and maintenance of hypertension is well-documented, involving pathophysiological mechanisms such as increased sympathetic nervous system activity, endothelial dysfunction, inflammation, oxidative stress, and insulin resistance [67]. Interventions addressing both smoking cessation and weight management are crucial. The recommendation for occupational health nurses to collaborate with management to strengthen health promotion programs is highly pertinent [52]. To mitigate the identified health risks, these programs should focus on supporting healthy lifestyles, including smoking cessation and improved nutritional habits, among hotel workers [60,68,69]. Such programs are particularly vital given that healthcare workers, despite their knowledge, often struggle with adopting preventive measures due to high-stress environments, a challenge that may extend to other demanding occupations such as hotel work [70,71].

In summary, this study effectively demonstrates that older age, overweight status, and smoking are associated with hypertension and leukocytosis among hotel workers in Indonesia. Because smoking behavior and nutritional status were measured using simplified categories, the observed associations likely underestimate the true variability in these exposures. More granular assessments, such as smoking pack per year, dietary intake measures, or metabolic indicators, are needed in future studies. Furthermore, beyond general nutritional status, investigating the specific types of dietary patterns contributing to overweight and hypertension could provide more actionable insights for interventions [10,72]. The study also provides a foundation for assessing the effectiveness of tailored health promotion programs within the hospitality industry, potentially leading to improved worker health outcomes and reduced incidence of NCDs. Despite these limitations, the findings of this study provide useful preliminary information for occupational health practice in hotel settings. The interpretation of these findings must also consider several methodological limitations. Important confounders, including physical activity, dietary intake, shift work, alcohol use, medication history, and preexisting illness, were not available in the dataset and could not be adjusted for. This creates the potential for residual confounding, which may influence the observed associations.

These findings highlight the influence of modifiable lifestyle factors and age-related physiological changes on cardiovascular and hematological outcomes in this occupational group. This study is consistent with global and national epidemiological data and underscores an intervention study for targeted and comprehensive occupational health practice to foster healthier lifestyles within this specific workforce. The results suggest that older workers, those with overweight status, and those who smoke may benefit from occupational health nurses’ targeted workplace health education and risk reduction initiatives. Future research could explore the long-term effects of smoking and nutritional status on the health of hotel workers by employing longitudinal designs to establish causality.

## 5. Limitations

This study provides valuable insights into the associations between smoking, nutritional status, hypertension, and hematological disorders among hotel workers in Indonesia, but it has several limitations inherent to its cross-sectional design and scope. The primary limitation stems from the cross-sectional nature of the study, which prevents the establishment of causal relationships. The measurement of exposure variables in this study is also limited. Smoking status was recorded only as a binary yes/no variable without information on smoking intensity, duration, pack years, or tobacco use type. As a result, we could not assess dose–response relationships or distinguish between occasional and heavy smokers. Similarly, nutritional status was assessed solely through BMI categories, which do not reflect body composition, fat distribution, dietary patterns, metabolic markers, or other nutritional determinants. Another limitation is related to the specific population studied: hotel workers in Indonesia. Although this provides insights into an occupational group, the findings may not be generalizable to other populations with different demographic profiles, socioeconomic statuses, occupational exposures, or genetic predispositions. While associations were identified, such as between older age (40–69 years) and overweight status with higher hypertension levels, and between smoking and increased leukocytosis, it is not possible to ascertain whether these factors directly cause the outcomes or if there are other unmeasured factors at play. Longitudinal studies are necessary to observe the temporal sequence of events and infer causality more robustly.

## 6. Conclusions

This study identified associations between older age, overweight nutritional status, and hypertension and between smoking and leukocytosis among hotel workers. Although these findings may inform occupational health monitoring and risk assessment, they do not imply causal relationships or predict the outcomes of potential interventions. Nevertheless, the results may offer useful preliminary insights for occupational health practice, suggesting that workers with modifiable risk factors, such as smoking and overweight status, could benefit from targeted health promotion activities within the workplace. Further research, particularly longitudinal studies with more comprehensive measurement of behavioral, clinical, and occupational exposures, is needed to confirm these associations and better understand the underlying pathways. Health promotion programs led by occupational health nurses, in collaboration with management, are crucial for fostering healthy lifestyles among hotel workers. To mitigate the risk of hypertension and hematological complications, these programs should include smoking cessation initiatives, weight management strategies, and educational campaigns on healthy eating. Addressing these modifiable risk factors can lead to improved cardiovascular health outcomes and enhanced overall well-being in this vulnerable occupational group, ultimately contributing to a healthier workforce and reducing the burden of NCDs in Indonesia.

## Figures and Tables

**Table 1:** Respondents’ characteristics, job level, and health criteria (nutritional, smoking habit, hypertension, and hematologic level) (N = 366).

Characteristic	Category	n (%)
Age	20–29	7 (1.9)
	30–39	113 (30.9)
	40–69	246 (67.2)
Gender	Male	316 (86.3)
	Female	50 (13.7)
Job Level	Cashier/Purchasing	50 (13.6)
	Housekeeping	30 (8.2)
	Security	32 (8.7)
	F and B	59 (16.1)
	Recreation/Pool	62 (16.9)
	Engineering/IT	37 (10.1)
	Front Office	21 (5.7)
	Admin/HR	13 (3.6)
	Ticketing	22 (6)
	Sales and marketing	8 (2.2)
	Manager	32 (8.7)
Nutritional Status	Underweight	6 (1.6)
	Normal	164 (44.8)
	Overweight	140 (38.3)
	Obesity	56 (15.3)
Smoker habit	Non-smoker	156 (42.6)
	Smoker	210 (57.4)
Hypertension	No	238 (65)
	Yes	128 (35)
Hematologic parameters	Leukocytosis	40 (10.9)
	Normal	290 (79.2)
	Anemia	36 (9.8)

**Table 2:** Effect of the respondents’ characteristics on their nutritional status and smoking habit.

Characteristic	Category	Hypertension		*p*-Value	Leukocytosis		*p*-Value	Anemia		*p*-Value
		No 238	Yes 128		No 326	Yes 40		No 330	Yes 36	
		(65%)	(35%)		(89.1%)	(10.9%)		(90.2%)	(9.8%)	
Age	20–29	7	0	<0.05	6	1	0.861	7	0	0.302
	30–39	87	26		102	11		105	8	
	40–69	144	102		218	28		218	28	
Gender	Male	200	116	0.08	280	36	0.475	287	29	0.287
	Female	38	12		46	4		43	7	
Job Level	Cashier/Purchasing	31	19	0.19	42	8	0.782	46	4	0.098
	Engineering/IT	20	17		34	3		34	3	
	F and B	42	17		51	8		53	6	
	Front Office	15	6		18	3		20	1	
	Housekeeping	24	6		29	1		30	0	
	Admin/HR	8	5		12	1		13	0	
	Manager	25	7		28	4		27	5	
	Recreation/Pool	34	28		58	4		53	9	
	Sales and marketing	8	0		7	1		7	1	
	Security	15	17		27	5		25	7	
	Ticketing	16	6		20	2		22	0	
Nutritional Status	Underweight	6	1.6	<0.05	155	19	0.739	158	16	0.400
	Normal	174	47.5		6	0		5	1	
	Overweight	139	38.0		122	17		122	17	
	Obesity	47	12.8		43	4		45	2	
Smoking habit	Non-smoker	94	62	0.09	146	10	<0.05	136	20	0.098
	Smoker	144	66		180	30		194	16	

Significance *p*-value calculated at α = 0.05.

**Table 3:** Effect of Age and Nutritional Status on Hypertension (HT).

Variables	B	S.E.	Wald	df	Sig.	Exp (B)	95% CI for EXP (B)	
							Lower	Upper
Age (years)	0.970	0.252	14.827	1	0.000	2.64	1.61	4.33
Nutritional status	0.312	0.099	9.980	1	0.002	1.37	1.17	1.66
Constant	−3.619	0.720	25.238	1	0.000	0.03		

Variable (s) entered: age and nutritional status; Significance (α) < 0.05.

**Table 4:** Effect of smoking status on leukocytosis.

Variable	B	S.E.	Wald	df	Sig.	Exp (B)	95% CI for EXP (B)	
							Lower	Upper
Smoking habit	−0.928	0.380	5.947	1	0.015	0.40	0.19	0.83
Constant	2.681	0.327	67.271	1	0.000	14.600		

Variable (s) entered: smoking habit. Significance (α) < 0.05.

## Data Availability

The original contributions presented in this study are included in the manuscript. Further inquiries should be directed to the corresponding author.
